# The determinants of COVID-induced brain dysfunctions after SARS-CoV-2 infection in hospitalized patients

**DOI:** 10.3389/fnins.2023.1249282

**Published:** 2024-01-08

**Authors:** Shahwar Yasir, Yu Jin, Fuleah A. Razzaq, Antonio Caballero-Moreno, Lidice Galán-García, Peng Ren, Mitchell Valdes-Sosa, Roberto Rodriguez-Labrada, Maria L. Bringas-Vega, Pedro A. Valdes-Sosa

**Affiliations:** ^1^Joint China-Cuba Laboratory for Neurotechnology and Bioengineering (JCCLNB), The Clinical Hospital of Chengdu Brain Science Institute, School of Life Sciences and Technology, University of Electronic Science and Technology of China, Chengdu, China; ^2^Servicio de Psiquiatria Galigarcia, Hospital Nacional, La Habana, Cuba; ^3^The Cuban Neuroscience Center, La Habana, Cuba

**Keywords:** SARS-CoV-2, neuropsychology, COVID-19, post-COVID neuropsychiatric symptoms/disorders, long-COVID

## Abstract

The severity of the pandemic and its consequences on health and social care systems were quite diverse and devastating. COVID-19 was associated with an increased risk of neurological and neuropsychiatric disorders after SARS-CoV-2 infection. We did a cross-sectional study of 3 months post-COVID consequences of 178 Cuban subjects. Our study has a unique CUBAN COVID-19 cohort of hospitalized COVID-19 patients and healthy subjects. We constructed a latent variable for pre-health conditions (PHC) through Item Response Theory (IRT) and for post-COVID neuropsychiatric symptoms (Post-COVID-NPS) through Factor Analysis (FA). There seems to be a potential causal relationship between determinants of CIBD and post-COVID-NPS in hospitalized COVID-19 patients. The causal relationships accessed by Structural Equation Modeling (SEM) revealed that PHC (*p* < 0.001) and pre-COVID cognitive impairments (*p* < 0.001) affect the severity of COVID-19 patients. The severity of COVID-19 eventually results in enhanced post-COVID-NPS (*p* < 0.001), even after adjusting for confounders (age, sex, and pre-COVID-NPS). The highest loadings in PHC were for cardiovascular diseases, immunological disorders, high blood pressure, and diabetes. On the other hand, sex (*p* < 0.001) and pre-COVID-NPS including neuroticism (*p* < 0.001), psychosis (*p* = 0.005), cognition (*p* = 0.036), and addiction (*p* < 0.001) were significantly associated with post-COVID-NPS. The most common neuropsychiatric symptom with the highest loadings includes pain, fatigue syndrome, autonomic dysfunctionalities, cardiovascular disorders, and neurological symptoms. Compared to healthy people, COVID-19 patients with pre-health comorbidities or pre-neuropsychiatric conditions will have a high risk of getting severe COVID-19 and long-term post-COVID neuropsychiatric consequences. Our study provides substantial evidence to highlight the need for a complete neuropsychiatric follow-up on COVID-19 patients (with severe illness) and survivors (asymptomatic patients who recovered).

## 1 Introduction

COVID-19 patients most frequently experience respiratory impairments. However, the disease’s high susceptibility and morbidity indicate the effect of pre-existing health conditions in COVID-19 patients. The degrees of symptomatology in COVID-19 are caused by host-viral interactions and immunological responses, leading to severe infection and mortality. We briefly summarized some of the literature that shows that patients with a history of diabetes, hypertension, or respiratory diseases have much worse immune function, which affects vascular and respiratory impairment (Huang et al., 2022; Sharun et al., 2022). Recent Research into COVID-19 revealed that SARS-CoV-2 affects the brain, which results in neurological and neuropsychiatric consequences. Indeed, after recovering from COVID-19, some patients still suffer from post-COVID symptoms, which include depression, anxiety, headache, sleep disturbances, cognitive decline, or other health comorbidities that can lead to long-COVID (Fahriani et al., 2021; Bigdelou et al., 2022, 2022; Efstathiou et al., 2022).

The risk of anxiety or trauma-related disorders is highest for people with pre-health comorbidities such as complicated diabetes, obesity, and cardiovascular diseases (Treskova-Schwarzbach et al., 2021). According to US administrative database data, 14% of individuals with SARS-CoV-2 infection in 6 months developed new clinical disorders affecting multi-organ systems, including cardiovascular, respiratory, or brain disorders (Daugherty et al., 2021). Another study of 4,899,447 hospitalized patients examined underlying medical conditions related to SARS-CoV-2 infection (Kompaniyets et al., 2021). Careful evaluation and management of underlying health conditions in COVID-19 patients can aid in risk stratification for severe infection (Calixto-Calderón et al., 2021). However, much is yet to be discovered about the detailed mechanisms, treatment, efficacy, and long-term outcomes of post-COVID symptoms (Adab et al., 2022; Choutka et al., 2022). Significant evidence shows that chronic conditions can develop from acute COVID-19, where hospitalization and pre-existing conditions are generally linked to higher risk and severity of COVID-19 (Cunningham et al., 2021).

There is increasing scientific evidence of an association between SARS-CoV-2 infection and the subsequent occurrence of new-onset post-COVID neuropsychiatric symptoms, especially among patients with increased disease severity (Charlton et al., 2021; Busatto et al., 2022; De Erausquin et al., 2022; Efstathiou et al., 2022). It is mandatory to understand the impact of the neurotropic nature of SARS-CoV-2 and its effects on the long-term consequences of cognitive decline and other neuropsychiatric sequelae in COVID-19 patients (Rogers et al., 2020; De Erausquin et al., 2022; Taquet et al., 2022). Of particular importance is that pre-existing neuropsychiatric disorders induce a higher risk of contracting COVID-19 infection (Douaud et al., 2022; Taquet et al., 2022). So, it is essential to understand the attributes of post-COVID or long-COVID symptoms in patients (Augustin et al., 2021; Fernández-de-Las-Peñas et al., 2021; Magnusson et al., 2022; Nalbandian et al., 2023).

Despite the numerous clinical manifestations of post-COVID sequelae, studies employing statistical approaches to analyze patterns of symptom co-occurrence and their biological connections explicitly are sparse. We leveraged a unique cohort obtained in Cuba to describe the long-term post-COVID consequences. It is a 3-month follow-up study on hospitalized COVID-19 patients and survivors after recovery. We have studied the potential causal relations between determinants of COVID-Induced Brain Dysfunctions, analyzing age, sex, pre-health conditions (PHC), pre-COVID neuropsychiatric conditions (Pre-COVID-NPS), COVID-19 severity, and long-term post-COVID neuropsychiatric symptoms (post-COVID-NPS) in hospitalized COVID-19 patients and healthy controls. The COVID-19-positive and COVID-19-negative patients were hospitalized and shared the same psychological stress. The risk/incidence for neuropsychiatric sequelae may increase with pre-existing health conditions, pre-neuropsychiatric conditions, and admission to the hospital for SARS-CoV-2 infection. This study identified the potential risk factors, including COVID-19 severity, the effect of pre-health comorbidities, and pre-neuropsychiatric conditions in patients. The uniqueness of this sample is that both PCR + and PCR- participants were hospitalized and were subject to some degree of isolation and stress.

The main goal is to check whether the patients with prior health comorbidities or pre-neuropsychiatric conditions have a high risk of a more severe COVID-19 infection, eventually leading to long-term post-COVID neuropsychiatric symptoms. So, we studied the effect of pre-health conditions (PHC) on COVID-19 severity. PHC includes chronic obstructive pulmonary disease, immunological disorders, renal disorders, high blood pressure, cardiovascular symptoms, and diabetes. Post-COVID-NPS analysis includes somatomorphic symptomatology and autonomic dysfunctionalities. The somatomorphic symptomatology includes pain, gastrointestinal symptoms, cardiovascular disorders, urogenital and neurological symptoms. Autonomic dysfunctionalities include fatigue syndrome, panic symptoms, symptoms of generalized anxiety, depression, sleep disorders, sexual dysfunctions, and trauma. Pre-health conditions were categorized and analyzed using Item Response Theory (IRT) by generating a latent variable. Symptom co-occurrence for post-COVID-NPS was investigated by Factory Analysis (FA). After adjusting for confounders (age, sex, and pre-COVID-NPS), we further explored the associations of latent variables with the long-term neuropsychiatric consequences in COVID-19 patients via Structural Equation Modeling (SEM).

## 2 Materials and methods

### 2.1 Study design

This study is a prospective follow-up study of convalescent COVID-19 subjects after epidemiological discharge who were recruited from community-based policlinics in three municipalities in Havana City. The assessment was conducted for 8 months, from July 2020 to March 2021. It included baseline assessments and a follow-up visit 18–24 months later. For this study, 178 participants were recruited. All Participants were asked to visit the Cuban Centre for Neurosciences for the health assessments.

### 2.2 Participants

#### 2.2.1 Inclusion and exclusion criteria

For this study, the SARS-CoV-2 infection cohort was selected retrospectively based on the following inclusion criteria: (i) age 18-80 years, (ii) at least primary school education, (iii) diagnosis of COVID-19 by Polymerase Chain Reaction (PCR) test; and (iv) after discharge period of 3–6 months. The exclusion criteria were (i) diagnosis of neurologic or psychiatric disorders before the COVID-19 infection, (ii) diagnosis of severe organ-specific disease (e.g., cancer, hepatopathy, cardiomyopathy, advanced renal disease), and (iii) alcohol or drug abuse.

Most of the patients analyzed in this study were infected between March and December 2020. They were evaluated between June and December 2020. The molecular epidemiological study conducted in Cuba identified that the common SARS-CoV-2 variant in Cuba during this time was D614G (Guzmán et al., 2022). However, a few COVID-19 cases were taken in early 2021 as well, which were infected with another variant of SARS-CoV-2. It is impossible to distinguish the variant of those few cases; however, the dominant variant of SARS-CoV-2 at that time was D614G.

#### 2.2.2 Patients

A total of 91 participants were confirmed convalescent COVID-19 patients who had contracted the D614G variant of the SARS-COV-2 virus and had a positive reverse transcription polymerase chain reaction (RT-PCR) result.

#### 2.2.3 Controls

To ensure the robustness of the study, we selected an RT-PCR negative control group (*n* = 87) comprising individuals of age, sex, and education-matched individuals. These controls were in close contact with COVID-19-positive cases. They were isolated and evaluated in healthcare facilities according to the “Cuban Protocol for the Management and Care of COVID-19 in 2020” until their PCR test results were available. Those who tested negative were discharged, while those who tested positive were shifted to a hospital for COVID-19 treatment. This control group was included to mitigate the potential impact of psychological determinants, such as anxiety and depression, on intergroup differences. The unique aspect of this study design allowed us to isolate the virus’s effects from other confounding factors. Including this control group is a noteworthy feature of the study, as it enhances the reliability and validity of the findings.

### 2.3 Measures/determinants of COVID-induced brain dysfunctions (CIBD)

#### 2.3.1 COVID-19 severity

The degree of severity for all the participants was classified into four categories. Non-COVID patients were coded as “0.” On the other hand, COVID patients (PCR positive) had distinct degrees of severity for SARS-CoV-2 infection. There were 44 asymptomatic patients coded as “1,” 37 mild symptomatic patients coded as “2,” and 10 severe symptomatic patients coded as “3.”

#### 2.3.2 Pre-health conditions (PHC)

We have analyzed seven different health categories to study the prior-health comorbidities in COVID-19 patients, which are as follows:

i **Cardiovascular diseases** include ischemia, stroke, hemorrhage, insufficient blood circulatory system issues, and arrhythmia.
ii **Immunological disorders** include immunodepression, immunosuppression, HIV, and rheumatoid arthritis.
iii **Renal disorders** include urinary sepsis, renal colic disorder, urinary incontinence, and kidney stones.
iv **Neurological diseases** include epilepsy, peripheral manifestations, aneurysm, migraine, multiple sclerosis, trigeminal neuralgia, craniofacial syndrome, neuropathy, and meningoencephalitis.
v **Chronic obstructive pulmonary disease (COPD)** includes lungs-related injury/disorder and fibrosis.
vi **High blood pressure (HBP).**
vii **Diabetes.**

#### 2.3.3 Confounders/confounding factors

##### 2.3.3.1 Age

Our study sample includes participants with an age range from 20 to 85 years. The mean age was 48.25 for the COVID-19 patients and 44.82 for the Control group. We treated age as a confounding factor to check its effect on other confounders (sex and pre-COVID-NPS) and COVID-19-related variables (PHC and post-COVID-NPS).

##### 2.3.3.2 Sex

We analyzed 36 males and 55 females in the COVID-19 group and 39 males and 48 females in the control group. It is to study whether sex (male or female) is associated with COVID-19 severity and post-COVID-NPS.

##### 2.3.3.3 Pre-COVID neuropsychiatric conditions (pre-COVID-NPS)

The psychiatrist’s team consisted of 4–6 psychiatrists from different hospitals in Cuba, who were in charge of interviewing the patients. The psychiatrists employed a few categories from Section 0 of the SCAN 2.1 (Schedules for Clinical Assessment in Neuropsychiatry), which includes sociodemographic items such as age, date of birth, sex, marital status, educational level, years of education, address, and skin color for a detailed analysis. Furthermore, they diagnosed the previous psychiatric conditions (Personal Pathological and Psychiatric Antecedents) after the interview, using different standardized screening questionnaires, and finally concluded their remarks to make a diagnosis.

The following are the categories of pre-COVID-NPS in COVID-19 patients.

**The Neurocognitive symptoms** category was based on three categories of syndromes: delirium, mild neurocognitive disorder, and major neurocognitive disorder (dementia). **The Psychotic spectrum** category classified all subjects referred to as having any psychotic symptom or episode. They are characterized by an impaired relationship with reality, usually associated with behavioral changes, such as hearing voices, visual hallucinations, or delusions. There are several psychotic disorders with different diagnostic criteria, as mentioned in the “Diagnostic and Statistical Manual of Mental Disorders” (DSM-5) 5th edition. **The Neuroticism spectrum** reflects a person’s level of emotional stability. It is usually defined as a personality trait with negative emotions, poor self-regulation (an inability to manage urges), trouble dealing with stress, and the tendency to complain. Non-psychotic symptoms, such as depression, panic, or anxiety episodes, were also classified. Lastly, **Addiction** includes those subjects who are referred to have abuse of substances. They have substance use disorder criteria that involve using more substance than usual, urges to use the substance often, neglecting responsibilities at home, work, or school because of intense cravings to use the substance, giving up social and extracurricular activities, and continual use of the substance despite causing problems in physical and mental health, etc.

Notably, Taquet et al. (2021) reported that among 236,379 patients diagnosed with COVID-19, the most common incidences at 6 months post-COVID consequences were 1.40% for psychotic disorders, 6.58% for substance use disorder, 5.42% for insomnia, and 0.67% for dementia.

##### 2.3.3.4 Post-COVID neuropsychiatric symptoms (post-COVID-NPS)

Neuropsychiatric data of COVID-19 patients was taken from Schedules for Clinical Assessment in Neuropsychiatry (SCAN), version 2.1 (Schützwohl et al., 2007). It is a semi-structured interview developed by the World Health Organization to assess psychiatric disorders that consists of 1,872 items distributed in 28 sections. For the present study, only sections 0 (sociodemographic items); II (Physical health, somatoform, and dissociative disorders), IV (Panic, anxiety, and phobias); VI (Depressed mood and ideation); VII (Thinking, concentration, energy, interest); VIII (Bodily functions), and XIII (Interference and attributions for part one) were analyzed. However, during the interview, the psychiatrists gathered additional information about the duration of symptoms, the initial and final date of symptoms, and interference of the symptoms, which helped make the diagnosis.

We have used Somatomorphic symptomatology items categorized by SCAN 2.1 (Schützwohl et al., 2007), which are defined as follows:

i **Pain** includes headache, pain in the back, arms, or legs, muscle pain, pain in the joints, pain in the chest, pain while urinating, abdominal pain, menstrual pain, pain during sexual intercourse, pain in the ass, pain in other parts of the body and erratic pain.
ii **Gastrointestinal** include nausea, vomiting, a sensation of fullness, bloating, diarrhea, constipation, anal fluids, bad sensation in the mouth, burning sensation in the chest or esophagus, aerophagia, hiccups, intolerance to some food, and other gastric symptoms.
iii **Cardiovascular** includes palpitation, precordial discomfort, hyperventilation, shortness of breath (without physical exercise), dyspnea during exercise, and other cardiovascular complaints.
iv **Urinary/Urogenital** include frequent urination, urinary retention, uncomfortable sensation around the urethra or genitalia, irregular menstruation, fluid in the vagina, excessive bleeding during menstruation, decrement of menstruation, vomiting during pregnancy, lack of sexual interest, erectile dysfunction, other complaints in the urinary and genital area.
v **Neurological** include lack of balance or equilibrium, a sensation of paresis or focal muscle weakness, swallowing problems, knot in the throat, aphonia, twinkling, painful anesthesia, diplopia, blindness, deafness, fainting, fading, loss of consciousness, amnesia, other neurological manifestations.

Of note, the neurological symptomatology included in our analysis is based on the participant’s answers asked during psychiatric assessments and is not actually from the neurological manifestations category defined by a neurologist. That is why, in our study, we have mainly focused on neuropsychiatric symptoms instead of neurological consequences. The complete list of the items included in this study can be found in Supplementary material 1.

## 3 Statistical analysis

**FIGURE 1:**
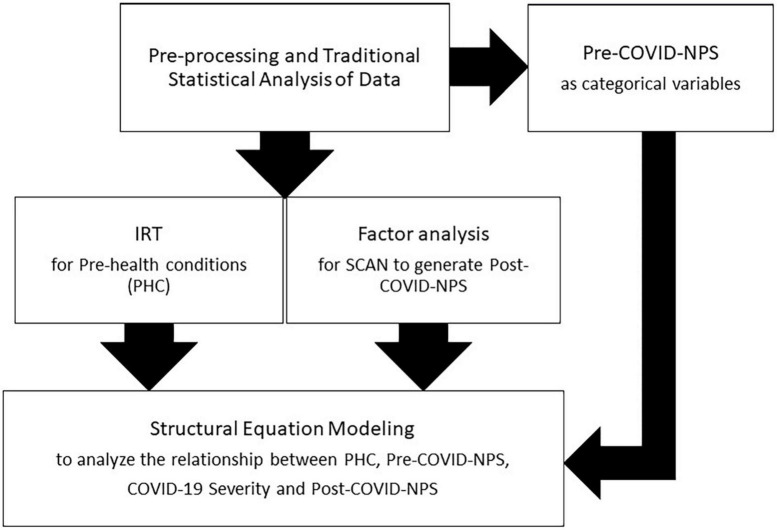
Overview of methodology (statistical analysis) for our CUBAN COVID-19 dataset.

This section describes the details of the statistical analysis for our data. Figure 1 shows an overview of the proposed methodology in this article.

### 3.1 Data pre-processing and cleaning

We pre-processed and cleaned the data and stored it in “tidyverse” format using the “tidyverse” package (Wickham et al., 2019) in R (version 4.3.0). To understand the data distribution, we have plotted individual data points in a Scatter plot between COVID-19 severity and post-COVID-NPS, available in Supplementary material 2 of the article. Furthermore, checking for linearity, additivity, normality, and homoscedasticity assumptions is important for any causal or inference-based analysis to determine the authenticity of the results. The violation of assumptions may degrade the outcome of the analysis (Williams et al., 2013; Ernst and Albers, 2017).

Initially, we check for data missingness, followed by multivariate outlier detection. A multivariate outlier is any subject whose combination of values for all variables differs from most participants (Tabachnick et al., 2007). We used Mahalanobis distance to assign a score to each subject based on the distance between the subject and the center of a distribution. We detected this using the “Mahalanobis” function in R with a *p*-value of 0.001 as a distance-cut off (Tabachnick et al., 2007). We examined normality, where all errors are generally distributed around zero using the standardized regression residuals. A histogram was plotted using the “moments” package (Komsta and Novomestky, 2022) to check the skewness and kurtosis in the data. Additivity was checked by looking into correlation for measured variables. The correlations were checked and plotted with the “corrplot” package (Wei and Simko, 2021). The homoscedasticity assumption is fulfilled when residuals have the same variance for all independent variable values. To check for homoscedasticity, we employed a scatter plot of standardized residuals vs. fitted values (Tabachnick et al., 2007). Power analysis was done to check the power of our sample by using “semTools” (Jorgensen et al., 2022) and “semPower” (Moshagen and Bader, 2023) packages in R.

### 3.2 Item response theory (IRT) for PHC

We obtained a latent variable for pre-health conditions (PHC) by Item response theory (IRT) as they are binary variables with YES/NO questions. IRT consists of mathematical tools that explain the relationship between latent variables and their observable values (true/false or multiple-choice questions). The goal of IRT in our analysis is to quantify the likelihood or tendency of a given response based on the relationship between trends of pre-health conditions in participants and the ability to distinguish characteristics of these items. Therefore, a dominant factor explaining most of the variance scores is expected.

Item Response Theory implies three assumptions: A unidimensional trait denoted by theta, the local independence of items, and a response to an item can be modeled by a mathematical item response function (IRF). In this study, the models predict the likelihood or probability of a correct response. One way to choose the best-fitted model is to assess its relative fit through its Information criteria. AIC estimates are compared, and the model with the lower AIC is chosen. It also helps to identify their optimal linear combination obtained by non-linear factor analysis to produce an inferred overall score, also called a latent variable. We used “MIRT,” a Multidimensional item response theory (Chalmers, 2012) package in R, to identify latent variables. The latent variables are independent of the evaluator and are more robust against fluctuations in score recording.

### 3.3 Factor analysis for post-COVID-NPS

Factor analysis (FA) is a data reduction tool that helps to reduce a large number of variables into a smaller number of components (factor) or latent variables. It removes any redundancy from a set of correlated variables in the data. As post-COVID-NPS is a continuous score from SCAN 2.1, we generated a latent variable for post-COVID-NPS with the help of factor analysis. This technique combines the highest common variances and projects them onto a common score. We use this score for further analysis to summarize all constituent variables.

### 3.4 Structural equation modeling (SEM) for statistical analysis

Structural equation modeling (SEM) is a comprehensive set of multivariate statistical techniques. This method combines component and multiple regression analyses to explore the structural relationship between measured and latent variables for testing different hypotheses (Beran and Violato, 2010). We used SEM to analyze the relationship between COVID-19-related variables and their effect on COVID-19 severity and post-COVID-NPS. SEM examines linear causal relationships among variables while accounting for measurement error. After getting a post-COVID-NPS latent variable from factor analysis, we analyzed the effect of age, sex, pre-health conditions, and pre-COVID-NPS on COVID-19 severity and post-COVID-NPS.

Structural Equation Modeling was performed with the help of “Lavaan,” defined as a latent variable analysis. Thus, SEM with Lavaan helps test path models with latent variables. Our model in SEM analyzed the direct effect of PHC on COVID-19 Severity. And how COVID-19 Severity enhances post-COVID-NPS chances even after adjusting for confounders. Statistical fit indices provide a way to quantify how well our model fits the data. The SEM model’s Comparative Fit Index (CFI) is the discrepancy function for any sample size. CFI ranges from 0 to 1, with a larger value indicating a better model fit. Acceptable model fit is indicated by a CFI value of 0.90 or greater. The Root Mean Square Error of Approximation (RMSEA) describes the residual in the model. RMSEA values range from 0 to 1, with a smaller RMSEA value indicating a better model fit. It is highly significant if *p* < 0.05 between the two nodes in the path model.

## 4 Results

### 4.1 Demographics

Table 1 shows the analyses of demographic characteristics of our CUBAN COVID-19 dataset with significant differences in age, sex, and year of education between the two groups. It was tabulated using the “table one” package (Yoshida and Bartel, 2022).

**TABLE 1:** The demographic characteristics of our CUBAN COVID-19 dataset.

	Description	COVID-19 group (Positive = 0)	Control group (Negative = 1)	*P*-value
*N* = Number of patients	178	91	87	–
Age [mean (SD)]	20–85 years	48.25 (12.68)	44.82 (12.83)	0.074
Sex [*n* (%)]	Male = 0	36 (39.6)	39 (44.8)	0.576
	Female = 1	55 (60.4)	48 (55.2)	
Education [mean (SD)]	Years of study/grade	14.10 (3.60)	14.79 (2.88)	0.158
Marital status [*n* (%)]	Divorced	8 (9.1)	7 (8.9)	0.943
	Married	24 (27.3)	21 (26.6)	
	Single	18 (20.5)	20 (25.3)	
	Union consensual	36 (40.9)	30 (38.0)	
	Widow	2 (2.3)	1 (1.3)	
Degree of severity [*n* (%)]	No COVID = 0	0 (0.0)	87 (100.0)	<0.001
	Asymptomatic = 1	44 (48.4)	0 (0.0)	
	Mild symptomatic = 2	37 (40.7)	0 (0.0)	
	Severe symptomatic = 3	10 (11.0)	0 (0.0)	

### 4.2 Data pre-processing and assumptions check

Our CUBAN COVID-19 dataset has no missingness. We implemented outlier detection based on Mahalanobis distance and excluded three subjects from the analysis based on the distance cut-off value. After outlier elimination, we have *n* = 175 participants to carry out the rest of the analysis with more authenticity, as shown in Table 2.

**TABLE 2:** Outlier detection via Mahalanobis distance in R.

Mahal < cutoff		
Mode	TRUE	FALSE
Logical	175	3

**FIGURE 2:**
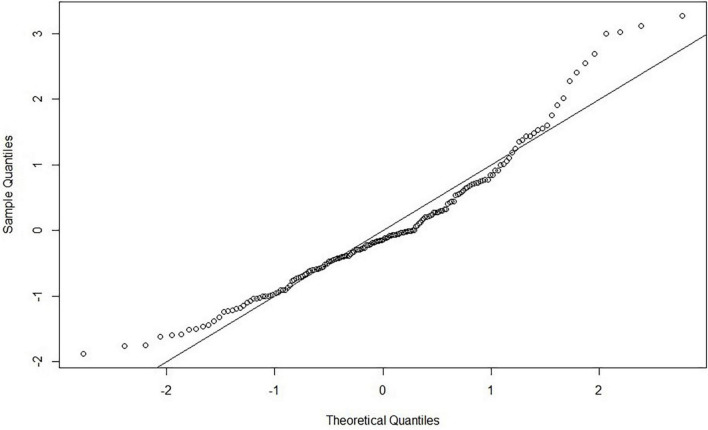
A Quantile-Quantile (Q–Q) plot for the standardized residual vs. a theoretical normal distribution to ensure a linear trend.

**FIGURE 3:**
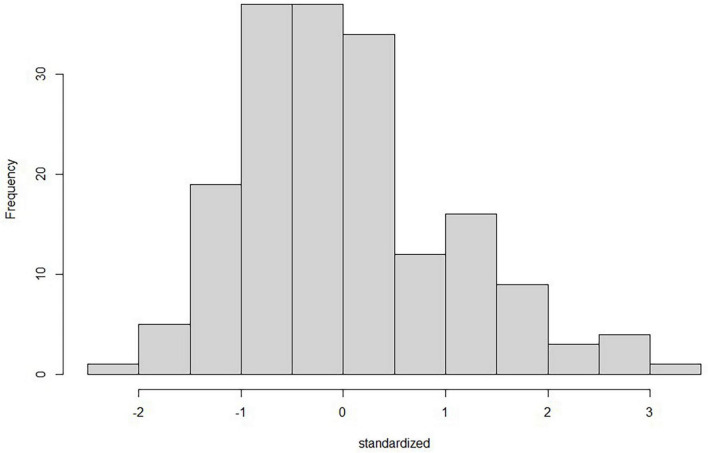
Histogram for standardized regression residuals to check for normality using the “moments” package in R. It shows that the data has a symmetrical distribution and is not skewed.

All the assumptions were checked and analyzed carefully by residual plots, histograms, and Quantile-Quantile (Q-Q) plots. Linearity was checked through a Q-Q plot where the *x*-axis has theoretical quantiles, showing the residuals in standard normal distribution if the variance is derived from a normal distribution. On the other hand, the *y*-axis has the sample quantiles for each data point. So, if the data have a normal distribution, it will be represented around a straight line. The recommended range is from –2 to + 2 for a linear relationship (Tabachnick et al., 2007). There is a minimal deviation in the tail, but overall, the Q-Q plot for standardized regression residuals shows a linear trend, as shown in Figure 2. Normality was checked and plotted as a Standardized Histogram to test whether residuals are normally distributed visually. The histogram for standardized residuals shows a normal data distribution (symmetrical in a bell shape), as shown in Figure 3, so no corrective measures were needed.

**FIGURE 4:**
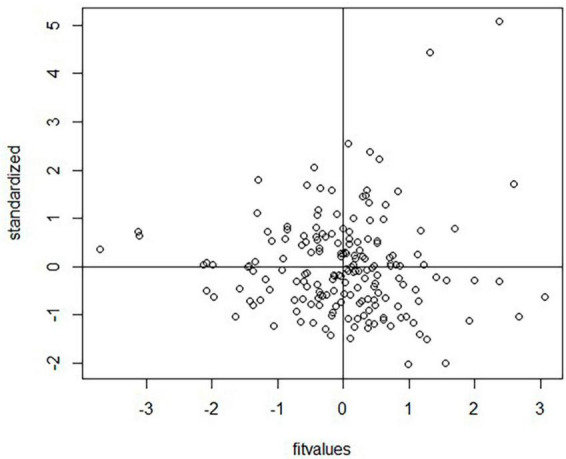
A homoscedasticity graph (scatter plot) between standardized regression residuals and z-scored fitted value where the data spread is homogeneous.

**FIGURE 5:**
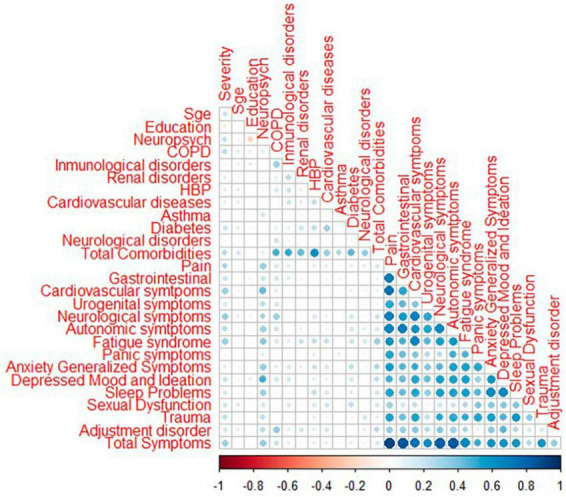
Correlation plot for measured variables using the “corrplot” package in R. The blue color shows a positive correlation; the dark color depicts a value of 0.96.

Furthermore, homogeneity/homoscedasticity was checked, depicting the residuals’ constant variance. We have plotted z-scores-fitted values vs. standardized residuals in a scatter plot. The data points have a constant spread along the regression line, as shown in Figure 4. The results are homogenous as the spread across both axes [−2, 2] is the same. The solid line in the plot marks zero for both axes. The additivity assumption was checked using “corrplot” to study the correlation between two independent variables (Wei and Simko, 2021). It shows that all the variables fall under the correlation value of less than 0.9, depicting a weak correlation/strength among variables, as shown in Figure 5. So, the analysis will not be biased, and the results will be accurate. The dots in Figures 2, 4 show the regression residuals as we are predicting the outcome of a random variable, so the errors should be randomly distributed (centered around zero). Each dot represents a standardized residual plotted against the theoretical residual for that area of the standardized distribution. By standardizing the errors, the results can be interpreted easily. In short, Figures 2–4 summarizes the results for linearity, normality, additivity, and data homogeneity using standardized results from regression.

### 4.3 IRT scores for pre-health conditions (PHC)

**FIGURE 6:**
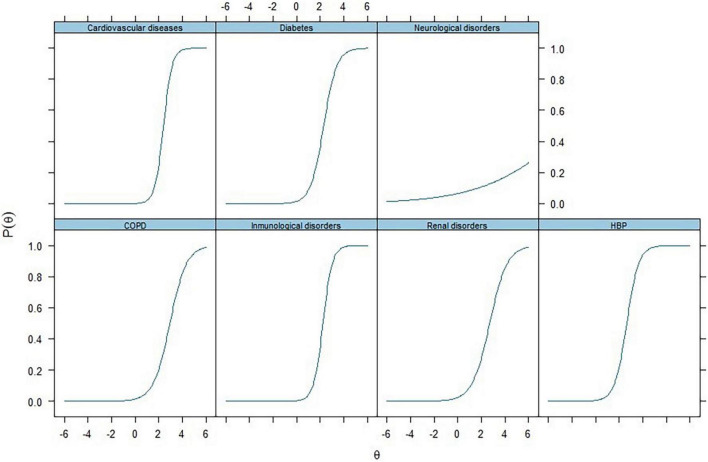
Item characteristic curves for pre-health conditions (PHC) via item response theory analysis (IRT). Each item’s response indicates a certain degree of loadings on the latent traits. Simply put, the latent variable (θ) influences and distinguishes the likelihood of reporting positive on the items in pre-health conditions.

We have analyzed pre-health comorbidities to get a latent variable for PHC by Item Response Theory, as IRT models estimate respondents’ responses to the items based on their position or trend on the latent trait spectrum. Each item’s response indicates a certain degree of loadings on the latent traits. Simply put, the latent variable (θ) influences and distinguishes the likelihood of reporting positive on the items in pre-health conditions. The Item Characteristic Curve is a graphical representation of this relationship. The curve is S-shaped (Sigmoid/Ogive), as indicated in Figure 6.

Furthermore, the higher the latent variable estimated, the higher the probability or likelihood of giving a positive response in terms of loadings. It is to be noted that theoretically, this latent variable (θ) ranges from -∞ to + ∞. However, practically, it usually ranges between −3 and + 3. This analysis was conducted with all 178 subjects, taking advantage of the flexibility of the IRT approach for handling missing values. We got specific loadings for pre-health conditions of COVID-19 patients through item response theory (IRT). The curves in Figure 6 show the probability of a patient being positive for values of an underlying variable, and the theta represents the outcome in the form of the disease state (COVID-19 severity and post-COVID-NPS). All items, specifically cardiovascular diseases, immunological disorders, and high blood pressure, loaded well onto the latent factors produced by IRT analysis with 47% of the variance, as listed in Table 3. It states that IRT classifies the participants suffering from any of these pre-health comorbidities to have a higher chance of getting a severe COVID-19 infection and post-COVID neuropsychiatric symptoms.

**TABLE 3:** Pre-health conditions (PHC) Scores from item response theory (IRT).

Pre-health conditions	Loadings
Cardiovascular diseases	0.852
Immunological disorders	0.833
High blood pressure (HBP)	0.759
Diabetes	0.731
Chronic obstructive pulmonary disease (COPD)	0.645
Renal disorders	0.626
Neurological diseases	0.157

### 4.4 Post-COVID neuropsychiatric symptoms (post-COVID-NPS) scores from FA

We did factor analysis for post-COVID-NPS and obtained factor loadings to describe the relationship between the factors and the observed variables. The loadings can evaluate the relationship strength between each variable and the factor. Additionally, we can identify the observed variables corresponding to a specific factor and interpret loadings as correlation coefficients. Values usually range from −1 to + 1. However, the sign indicates the direction of the relations, either positive or negative, while the absolute value indicates the strength. Stronger relationships in the factor analysis have factor loadings closer to −1 and + 1, which shows that the factors explain maximum variance in the observed variable. We have set the threshold to be >0.3 to check the significant values. For post-COVID-NPS, the highest loadings observed are for pain, fatigue syndrome, autonomic, cardiovascular, and neurological symptoms, with a threshold range from 0.82 to 0.74, explaining 47% of the variance, as indicated in Table 4.

**TABLE 4:** Post-COVID-NPS scores from factor analysis.

Post-COVID-NPS symptoms	Loadings
Pain	0.82
Autonomic symptoms	0.78
Cardiovascular symptoms	0.77
Fatigue syndrome	0.74
Neurological symptoms	0.74
Sleep problems	0.73
Anxiety generalized symptoms	0.73
Depressed mood and ideation	0.72
Gastrointestinal symptoms	0.68
Trauma-related symptoms	0.66
Panic symptoms	0.57
Urogenital symptoms	0.55
Adjustment disorder	0.53
Sexual dysfunction	0.38

### 4.5 Structural equation modeling (SEM) output

**FIGURE 7:**
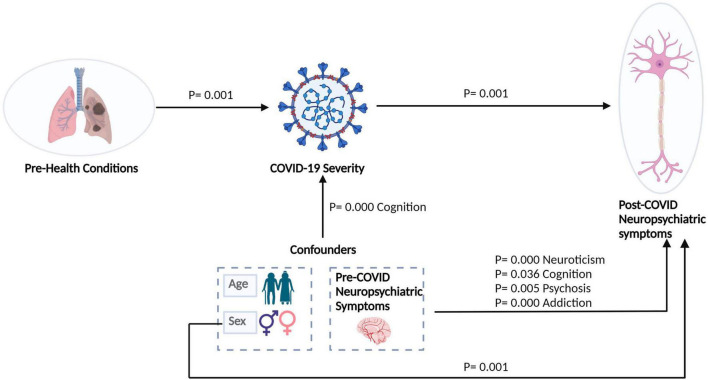
Relationship of COVID-19 with post-COVID-NPS of COVID-induced brain dysfunctions (CIBD) shown by structural equation modeling (SEM). A circle denotes a latent variable. A square denotes a measured variable. Directed arrows denote putative causal relations.

Structural Equation Modeling is a multivariate, hypothesis-driven technique based on a structural model. It represents a hypothesis about the causal relations among several variables. Path models are diagrams in SEM used to visually display the hypotheses and help to examine variable relationships. The relationship between constructs (variables that are not directly measurable) and their assigned indicators is depicted by arrows. Single-headed arrows have predictive relationships and can be interpreted as causal relationships, as shown in Figure 7.

The psychiatrists analyzed pre-neuropsychiatric conditions (Pre-COVID-NPS). We treated them as categorical variables as they belong to four different categories to study the effect of pre-COVID-NPS on post-COVID-NPS. The pre-COVID-NPS items were directly put in the Structural equation modeling formula along with other regression coefficients and confounding factors. SEM analysis helps to study the effect of these COVID-19 determinants of COVID-Induced Brain Disorders on post-COVID neuropsychiatric symptoms in COVID-19 patients. Significant and non-significant pathways (*p*-values) were obtained for latent variables and confounding factors to study the strength of their relationship, as shown in Table 5. The specific linear regressions tested are expressed using the Wilkinson-Rogers notation, which gives a compact and intuitive notation for liner models (Wilkinson and Rogers, 1973) as shown in Equations (1, 2). Wilkinson notations describe regression and repeated measure models without mentioning coefficient values. It also specifies and identifies the response variable as well as which predictor variables should be included or excluded from the model. So, it allows the inclusion of only the interaction terms of interest in the main model, be it squared, higher order terms, or grouping variables.


(1) Severity∼PHC+Age+Sex+Neuroticism+

Cognitive+Psychosis+Addiction

(2) NPS∼COVID-19⁢Severity+Age+Sex+Neuroticism+

Cognitive+Psychosis+Addiction

NPS=Post-COVID-NPS

Neuroticism+Cognitive+Psychosis+Addiction

=P⁢r⁢e-C⁢O⁢V⁢I⁢D-N⁢P⁢S

**TABLE 5:** Effect of COVID-19 severity and post-COVID-NPS on age, sex, and pre-COVID-NPS via structural equation modeling (SEM).

CIBD determinants	Estimate	*P*(> | z|)
**Severity∼**		
PHC	0.293	0.003**
Age	0.008	0.143
Sex	0.008	0.955
Neuroticism	0.006	0.534
Cognitive	2.051	0.000***
Psychosis	0.684	0.266
Addiction	0.821	0.187
**NPS∼**		
Severity	0.236	0.000***
Age	−0.001	0.775
Sex	0.413	0.001**
Neuroticism	0.667	0.000***
Cognitive	1.071	0.036*
Psychosis	1.637	0.005**
Addiction	2.285	0.000***

*p < 0.05,

**p < 0.01,

***p < 0.001.

The results summarize the factors affecting COVID-19 severity and post-COVID-NPS tested via Structural Equation Modeling (SEM). The output of the SEM model has significant fit indices. The comparative fit index, or CFI, is a popular fit index, which is 0.927 for our model. On the other hand, SRMR is 0.022, and RMSEA = 0.186, which shows that our model is a good fit with maximum loadings for latent variables. We obtained significant paths and *p*-values for sex, COVID-19 severity, PHC, pre-COVID-NPS, and post-COVID-NPS. For PHC and severity *p* = 0.005, for severity and NPS *p* < 0.001, for sex and post-COVID-NPS *p* < 0.001, and for pre-COVID-NPS (neuroticism, cognitive, psychosis, addiction) to post-COVID-NPS *p* < 0.05 as shown in Table 5.

The present study aims to assess the true extent of post-COVID brain dysfunctions in the Cuban population and its role in the progression or *de novo* diagnosis of neurodegenerative diseases. It depicts that COVID-19 is associated with a certain degree of severity and post-COVID neuropsychiatric symptoms (Post-COVID-NPS) in patients. Patients with pre-health comorbidities, like chronic obstructive pulmonary diseases, diabetes, immunological disorders, renal disorders, or cardiovascular symptoms, have a high risk of getting more severe COVID-19 and, eventually, more long-term neuropsychiatric symptoms. The most common neuropsychiatric symptoms included pain, cardiovascular, trauma, fatigue syndrome, anxiety, depression, and neurological symptoms. A study by Tauqet et al. has also shown that pre-COVID-NPS conditions enhance the chances of getting post-COVID-NPS comorbidities in COVID-19 patients.(Taquet et al., 2022).

## 5 Discussion

In this study, we used statistical methods to document patterns of co-occurrence of several post-COVID symptoms in a relatively moderate sample of patients with mild or severe COVID-19 infection. The participants were evaluated 3 months after hospitalization to determine how such a symptom co-occurrence was related to COVID-19 severity, followed by signs of post-COVID neuropsychiatric symptoms in patients and survivors (both hospitalized). Evidence from COVID-19 infection suggests a potential development and risk of long-term neuropsychiatric disorders (Kumar et al., 2021). We analyzed the pre-health conditions of COVID-19 patients and controls by Item Response Theory (IRT) and got PHC scores in the form of loadings. Another study illustrates and emphasizes the features of IRT to refine and increase the validity and reliability of the analysis (Zanon et al., 2016). In our study, the highest loadings (cardiovascular, immunological disorders, and high blood pressure) imply that the patients with such prior health comorbidities will have a high level of COVID-19 severity. However, neurological diseases in PHC loaded extremely low as our sample has few patients who had neurological disorders before getting SARS-CoV-2 infection. Similar other studies state that pre-existing health comorbidities may cause disease severity or post-COVID-related consequences (Sanyaolu et al., 2020; Calixto-Calderón et al., 2021; Edison, 2021a; Richardson et al., 2021; Treskova-Schwarzbach et al., 2021; Russell et al., 2023). Several underlying diseases were linked to severe COVID-19 infection. The most common condition was high blood pressure, but the significant risk factors for severe COVID-19 were obesity, diabetes with other comorbidities, and psychological disorders (Kompaniyets et al., 2021).

On the other hand, pre-COVID-19 neuropsychiatric conditions directly impact the severity of the infection and post-COVID neuropsychiatric symptoms. Patients with cognitive impairments prior to infection are more likely to get severe COVID-19 (Kumar et al., 2021). However, prior neuropsychiatric conditions like neuroticism, psychosis, addiction, and cognitive impairments significantly affect post-COVID-NPS in our study sample. Few neuropsychiatric manifestations of COVID-19 may cause severity in post-COVID neuropsychiatric symptoms in patients still recovering from SARS-CoV-2 infection and even those who recovered once and yet have some late health consequences (Charlton et al., 2021; Sampogna et al., 2022). COVID-19 increases the risk for neuropsychiatric sequelae, including psychosis (Chacko et al., 2020). An altered mental state is one of the most common neuropsychiatric conditions directly impacting effect, behavior (e.g., agitation), and cognition (Helms and Meziani, 2020). A study by Taquet et al. showed that there are many psychiatric conditions, such as bipolar disorder (BPD), major depressive disorder (MDD), panic disorder (PD), generalized anxiety disorder (GAD), or post-traumatic stress disorder (PTSD), and cognition disorders such as Alzheimer’s disease which could be worsened by the COVID-19 infection or fear of getting an infection (Taquet et al., 2021).

We treated age, sex, and pre-COVID-NPS as confounders to check the effect of COVID-19 severity on post-COVID-NPS. Even after adjusting for confounders, we still got a significant *p* < 0.001 value between COVID-19 severity and post-COVID-NPS. See preliminary results at Yasir et al. (2023). Another study also provides evidence that COVID-19 infection is associated with a significant risk of various autoimmune diseases, such as rheumatoid arthritis, sclerosis, etc., in COVID-19 patients. They have analyzed the study with demographic covariates, age, sex, race, socioeconomic determinants of health, lifestyle-related problems, and health comorbidities (Type 2 diabetes mellitus, depression, chronic kidney disease, sleep disorder, and psychoactive substance use) in different ethnicities (Chang et al., 2023).

Poor general health, pre-pandemic mental health, and sociodemographic characteristics have emerged as significant determinants in various COVID-19 patient studies (Stefano et al., 2022). Symptoms related to fatigue, dyspnea, headache, and anosmia were characterized as long-COVID conditions in patients and were more likely severe with increasing age, BMI, and female sex (Sudre et al., 2021). Different factors affect COVID-19 severity and post-recovery symptoms in patients. Female sex, increasing age, obesity, smoking, vaping, hospitalization with COVID-19, and being a healthcare worker were directly associated with a higher probability of persistent symptoms (Whitaker et al., 2022). However, in our case, age is not directly relevant to the severity of COVID-19 infection or post-COVID consequences because we have treated it as a confounder. A previous study reported that people who had recovered from COVID-19 with no longer reporting symptoms still experienced significant cognitive deficits while controlling for age, sex, education, pre-existing medical disorders, tiredness, depression, and anxiety (Hampshire et al., 2020).

There is a large body of evidence that COVID-19 affects the brain; it may infect and damage specific brain cells directly, resulting in memory loss, strokes, and other brain-related disorders (Edison, 2021b; Marshall, 2021; Nalbandian et al., 2021). Past studies have shown that many COVID-19 patients have experienced COVID-Induced Brain Dysfunction (CIBD) 6 months to 1 year after recovery (De Berardis et al., 2022). Apart from direct CIBD resulting from SARS-CoV-2 infection, prolonged pre-health conditions, disease-associated factors, work-related stress, social distancing, and quarantine (isolation) have affected the mental health of many infected and recovered people. Therefore, complete follow-up on COVID-19 patients (critically ill or recovered) is mandatory to lessen the effects of long-CIBD (Valdes-Sosa et al., 2021). Post-COVID-NPS analysis shows that critically ill or severe patients, after recovery, may still have few complications related to neuropsychiatric symptoms. Our study’s healthy controls experienced some post-COVID neuropsychiatric symptoms like anxiety and depression due to isolation and quarantine. In our sample size, the highest loadings obtained from factor analysis for post-COVID-NPS are for pain, autonomic dysfunctionalities, cardiovascular disorders, fatigue syndrome, and neurological symptoms, with a threshold ranging from 0.74 to 0.82. However, in this study, we have not classified or differentiated the origin of the post-COVID-symptoms, i.e., fatigue syndrome (organic or immunological) and restricted our analysis to behavioral measures. We are working on more biological variables in a further publication by studying the quantitative electroencephalogram (qEEG), which is based on a causal analysis to check qEEG as a proxy for brain functions that mediate the effect of Post-COVID-NPS in COVID-19 patients. Preliminary results showed that qEEG is a mediator and can be a biomarker to understand the effects of COVID-19 severity on post-COVID neuropsychiatric symptoms (Jin et al., 2023). On the other hand, a recent paper by Tauqet et al. studied blood biomarkers in 1837 COVID-19 patients to analyze the cognitive brain deficits where fatigue is the mediator for the biomarker profile, which clearly states that it is linked with post-COVID neuropsychiatric (cognitive) consequences in COVID-19 patients (Taquet et al., 2023).

COVID-19 infection, especially long-COVID, is associated with increased short and long-term risks of cardiovascular diseases in recovered patients (Chilazi et al., 2021). A follow-up on signs and symptoms of developing these cardiovascular complications post-COVID diagnosis or recovery up to a year or two may benefit infected patients with severe illness (Inciardi et al., 2020; Ramadan et al., 2021; Wan et al., 2023). Douaud et al. (2022) carried out one of the largest studies on COVID-19 and compared two groups (COVID and non-COVID patients). They found several significant longitudinal effects, including a severe reduction in the texture of gray matter. Moreover, SARS-CoV-2-infected individuals displayed a larger average cognitive impairment between the two-time points (Douaud et al., 2022). A recent study of one of the largest cohorts (*n* = 1733) on COVID-19 patients reported that at 6 months after symptom onset, patients discharged from the hospital or recovering from COVID-19 still have symptoms of fatigue, including muscle weakness, sleep difficulties, and anxiety or depression (Bourmistrova, 2022). Around 68% of patients reported at least one symptom, and such patients can be a primary target population for a follow-up and long-term recovery protocol (Huang et al., 2023). Two studies showed that the most frequent neuropsychiatric symptoms were sleep disturbance/disruption, followed by fatigue, objective cognitive impairment, anxiety, and post-traumatic stress (Badenoch et al., 2022; Sampogna et al., 2022), as analyzed in our results.

SARS-CoV-2 infection is commonly associated with a wide range of persistent, long-lasting symptoms, now known as post-COVID or long-COVID (Choutka et al., 2022). Many studies (Davis et al., 2021, 2023; Al-Aly et al., 2022) from the past determine which symptoms are associated with confirmed SARS-CoV-2 infection (post-recovery) in non-hospitalized individuals, as well as the risk factors for developing long-lasting symptoms in COVID-19 patients. Most frequent long-COVID symptoms include anosmia, shortness of breath at rest, chest pain, obesity, and other comorbidities such as COPD (Subramanian et al., 2022). Neuropsychiatric symptoms were common up to 5–6 months after initial hospitalization in hospitalized patients and recovered patients with no symptoms (Castro et al., 2021). A systematic review of psychiatric and neuropsychiatric sequelae of COVID-19 patients states that post-COVID symptoms such as post-traumatic stress disorder (PTSD), cognitive deficits, and sleep disturbances are persistent after hospital discharge till 7–9 months (Renaud-Charest et al., 2021). Risk factors associated with COVID-19 were disease severity and duration of symptoms (Schou et al., 2021). Kyzar et al. (2021) reported findings from a study of 52 patients recruited from New York City following acute COVID-19 infection and found a high level of correlation between psychiatric symptoms in participants. Many participants had clinically significant insomnia, depression, and post-traumatic stress at follow-up compared to baseline, indicating that post-COVID neuropsychiatric symptoms may grow over time (Kyzar et al., 2021).

As indicated earlier, fatigue, muscle weakness, sleep difficulties, and anxiety or depression were common, even 6 months after symptom onset (Badenoch et al., 2022). This result is consistent with studies from previous SARS long-term follow-ups in patients. Canadian researchers found that most SARS survivors had good physical recovery after illness, but 1 year later, 33% reported a significant decline in mental health. A follow-up study of SARS survivors showed that 40% of patients still had a chronic fatigue problem for a mean period of 41⋅3 months after SARS infection (Tansey, 2007; Lam, 2009). Similarly, our study analyzes the post-COVID-NPS in COVID-19 patients, which can help us better understand the long COVID-19 symptoms in future Research. The causal association between COVID-19 severity and post-COVID-NPS was studied through SEM, and the hypothetical causal relations are based on anatomically plausible connections between them. The strength of each association is specified by a path coefficient, which, by analogy to a partial regression coefficient, indicates how the variance of one region depends on the variance of another region if all other influences are held constant.

Long-COVID or post-COVID syndrome is poorly understood because it affects COVID-19 survivors at all disease severity levels, including younger people, children, and those not hospitalized (Bourmistrova et al., 2022; Frontera and Simon, 2022). Notably, neuropsychiatric deficits in long-COVID have been mainly associated with ACE2-rich brain areas, affecting inflammatory, metabolic, and degenerative processes (Guedj, 2021). The most typical symptoms following acute COVID-19 (long-COVID) are cognitive and mental disabilities, heart palpitations, fatigue, cough, headache, gastric, and cardiovascular diseases, all possible long-term effects (Efstathiou et al., 2022). Female sex and prior psychiatric problems can be risk factors for getting long-term COVID-19, although additional study is needed to support such risk factors (Yong, 2021). However, there are a few factors that contribute to the heterogeneity in long-COVID prevalence estimates, such as disparities in vaccinations, SARS-CoV-2 variants, pre-existing health comorbidities, study sample size, and use of varying non-COVID-19 control groups bring bias and appear to drive heterogeneity in prevalence estimates of long-COVID (Raman et al., 2022). Nevertheless, these factors help analyze the risk factors associated with long-term post-COVID symptoms (long-COVID). Our study has a small data set, but patients with any pre-health or pre-neuropsychiatric conditions have more chances of severe COVID-19 with enhanced post-COVID neuropsychiatric disorders. It can help us better evaluate long-COVID symptoms in late COVID-19 stages of recovered patients, as indicated in Figure 7. The output of the Lavaan Model for our hypothesis is as follows:

• Lavaan Model found that PHC and Pre-COVID-NPS affect the severity of SARS-CoV-2 infection that eventually affects post-COVID-NPS in COVID-19 patients.
• Pre-COVID-NPS (neuroticism, cognitive impairments, psychosis, addiction, and sex directly affect post-COVID-NPS in COVID-19 patients). It is to be noted that in our sample, the female sex is more significantly related to post-COVID-NPS rather than the male. The reason could be that few post-COVID neuropsychiatric symptom categories/sub-items from SCAN 2.1 are more related to female problems/disorders. On the other hand, Pre-COVID-NPS (specifically cognitive impairments) has a significant *p*-value, directly affecting the severity of COVID-19. So, people with cognitive impairment issues may face more severe COVID-19 symptoms.
• Even after controlling for confounders such as age, sex, and pre-COVID-NPS, the severity of COVID-19 is still significantly associated with post-COVID-NPS.

### 5.1 Summary

Early in the pandemic, concerns about the neurological and neuropsychiatric outcomes in COVID-19 patients and survivors were raised. Understanding the effect of SARS-CoV-2 infection and the fear of getting an infection (subject to isolation) on the brain remains unclear despite all the clinical investigations available. The significant gap lies in the mid to long-term neuropathogenic effects of the SARS-COV-2 infection due to the wide variety of mechanisms of viral entry into the brain.

The present study protocol is a longitudinal study of brain disorders in Cuban patients and survivors of COVID-19. It aims to characterize the post-COVID brain dysfunctions in all hospitalized subjects (patients and controls). The healthy subjects (controls) were initially hospitalized for being in close contact with the COVID-19 positive patients and later discharged upon having negative PCR reports. The general result of our analysis depicts that according to psychiatric assessments in SCAN 2.1 (Schützwohl et al., 2007), COVID-19 illness, along with a level of severity, leads to psychiatric manifestations. The hospitalized COVID-19 convalescents/patients showed higher somatomorphic symptomatology and autonomic dysfunctionalities than the healthy controls/non-COVID-19 patients. The convalescence phase of COVID-19 is characterized by a varied spectrum of specific neuropsychiatric manifestations that are intensified in subjects with psychiatric histories or pre-health comorbidities. It will eventually increase the incidence of post-COVID-related neuropsychiatric symptoms.

We created a latent variable combining all the prior-health symptoms using Item Response Theory (IRT), a statistical approach suitable for scaling numerous health outcomes along a single severity continuum (latent trait modeling). This latent variable was used further to investigate the relationship with post-COVID-neuropsychiatric symptomatology in COVID-19 patients. Finally, we studied the association of the latent variables with COVID-19 severity and post-COVID-NPS through Structural equation modeling. Compared with other studies on brain disorders in long-COVID, our study has some strengths that make it unique. The first advantage is the homogeneity in the clinical management of the cases because Cuba uses a single medical action protocol for the whole country so that the variability in the treatment and other medical procedures will be reduced. In addition, we studied a control group of people with negative PCR who were isolated in hospital institutions and were contacts of confirmed cases. It will minimize the intergroup differences explained by the negative psychological effect associated with the receipt or suspicion of a positive PCR. Another advantage is that our study involves a face-to-face evaluation of the subjects, promoting safer and more objective data collection compared to other studies conducted online or through telephone questionnaires. We have been powered to evaluate whether factors such as sex, pre-existing health comorbidities, admission to hospital because of COVID-19, and pre-neuropsychiatric conditions modify the risk of long-term neuropsychiatric sequelae after the acute infection. Even after adjusting for confounding factors like sex, age, pre-health conditions and pre-neuropsychiatric conditions, the most common post-COVID-neuropsychiatric symptoms in our study include pain, fatigue syndrome, autonomic dysfunctionalities, cardiovascular disorders, and neurological symptoms. The recurrence of these post-COVID symptoms is supported by many other studies stated in the discussion session of this article. These findings support the idea that there is a significant degree of co-occurrence of multiple post-recovery symptoms associated with pre-health and pre-neuropsychiatric conditions in COVID-19 patients assessed several months after hospitalization, implying that common underlying pathological mechanisms may influence the persistence of long-term post-COVID-neuropsychiatric consequences.

### 5.2 Limitations

The current sample size shows strong and reliable results for COVID-19 patients to have more post-COVID consequences if they have prior health comorbidities. However, the sample size is small and biased as few patients have severe symptomatic consequences after COVID-19. It is a cross-sectional rather than a longitudinal study, so the results need further validation. The statistical power of our sample is 0.984 for an effect size of 0.7 and 0.999 for an effect size of 0.5, respectively.

On the other hand, SARS-CoV-2 is a virus affecting the immune system. However, we didn’t use any immunological quantitative measures or other biomarkers to distinguish between the origin of organic and psychological symptoms. We only focused on the subjective reports by COVID-19 patients and neuropsychiatric assessments by the psychiatrists.

### 5.3 Possible future direction

The severity of COVID-19 is affected by many factors that may lead to long-term post-COVID-NPS. Long-COVID is a multisystemic illness that impacts multiple organ systems in the human body. There are many potential risk factors associated with long-term neuropsychiatric sequelae of COVID-19. Older age, pre-health status, and psychological factors significantly enhance the severity of COVID-19 and its consequences. They might trigger either the acute onset of neuropsychological manifestations or the worsening of the existing neuropsychiatric conditions. So, future research is needed to lessen the chances of getting severe COVID-19 or post-COVID-NPS in critically ill or recovered patients.

## 6 Conclusion

Patients with pre-health comorbidities and pre-neuropsychiatric conditions have a high risk of getting more severe COVID-19. It demonstrates how the severity of COVID-19 had a causal relationship with post-COVID neuropsychiatric symptomatology, even after adjusting for confounders, i.e., age, sex, and pre-COVID-19 neuropsychiatric symptoms. So, our results present evidence from a small but controlled cohort explaining that neuropsychiatric symptoms may worsen over time, particularly in COVID-19 patients and survivors with prior health comorbidities and neuropsychiatric disorders.

On the other hand, our study has a valuable sample of those COVID-19 subjects who were hospitalized at the same time as PCR-positive patients but later got negative PCR tests and were discharged. However, the sample suffered from the same emotional and psychological impact as the COVID-19 patients due to hospitalization, isolation and other environmental factors, thereby exhibiting a unique sample to eliminate the confounding variables from the analysis and isolate the COVID-19 effects. Future studies should continue to investigate and follow up in broader populations while exploring the potential mechanisms that may help to understand the neuropsychiatric pathology after SARS-CoV-2 infection.

## Data availability statement

The dataset supporting the conclusions of this article will be made available by the co-authors affiliated with Servicio de Psiquiatria and The Cuban Neuroscience Center upon reasonable request.

## Ethics statement

The studies involving humans were approved by the Local Research Ethics Committee of the Cuban Centre for Neurosciences and the National Hospital “Enrique Cabrera.” approved this study. All eligible participants were informed of the research purpose and confidential data processing, and they signed the consent form before the beginning of the experiments. All procedures were according to the Helsinki Declaration. The patients/participants provided their written informed consent to participate in this study. The studies were conducted in accordance with the local legislation and institutional requirements. Written informed consent for participation in this study was provided by the participants’ legal guardians/next of kin.

## Author contributions

SY and YJ curated the CUBAN COVID-19 data and carried out statistical analyses. SY worked on the clinical data/analysis and YJ is working on the EEG analysis in another manuscript. PV-S directed the study with FR and MB-V including formulating the hypotheses, selecting the research approach, supervising results, and revising the manuscript. FR guided statistical analysis. RR-L, AC-M, PV-S, and MV-S designed the Cuban cohort study, with RR-L and AC-M directing the data collection. LG-G contributed to the organization and curation of the databases. PR gave valuable suggestions for data analysis and manuscript revision. SY wrote the original draft of the manuscript and worked on reviews and revisions. All authors contributed and approved the submitted final version of the article.
