# Supplementary figures and images for: The determinants of COVID-induced brain dysfunctions after SARS-CoV-2 infection in hospitalized patients
Source: Front Neurosci. 2024 Jan 8;17:1249282. doi: 10.3389/fnins.2023.1249282 (PMC10800467; doi:10.3389/fnins.2023.1249282)

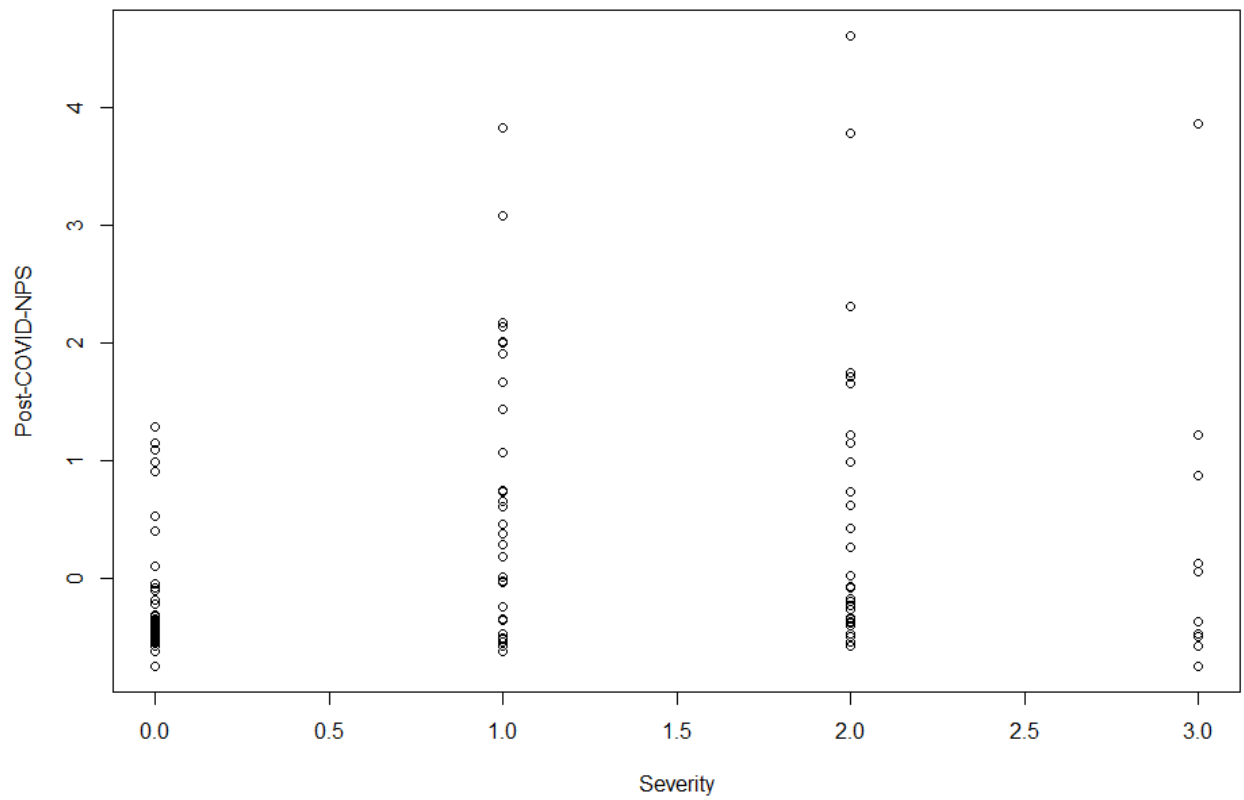

**Scatter plot between COVID-19 Severity and Post-COVID-NPS to plot individual data points.**

Supplement: Supplementary file 2 [file Data_Sheet_1.PDF]
